# REGARDS Cognitive Assessment and Approaches to Defining Cognitive Impairment and Change in Cognitive Function

**DOI:** 10.1093/geroni/igaa057.3152

**Published:** 2020-12-16

**Authors:** Jennifer Manly, Frederick Unverzagt, Leslie McClure, Suzanne Judd, J David Rhodes, George Howard

**Affiliations:** 1 Columbia University, New York, New York, United States; 2 Indiana University School of Medicine, Indianapolis, Indiana, United States; 3 Drexel University, Philadelphia, Pennsylvania, United States; 4 University of Alabama at Birmingham, Birmingham, Alabama, United States

## Abstract

Since 2003, REGARDS participants have taken part in telephone-based cognitive assessments. Global cognitive status is assessed annually with the Six-item Screener. Between 2006 and 2009, measures of learning and memory (CERAD Word List) and language/executive function (Animal and Letter Fluency) were implemented, and are administered biennially. A Brain Health Substudy, conducting in-home clinical examinations of neuropsychological, neurological, and functional status among 1000 participants, is underway to validate telephone assessments and estimate prevalence of VCID in REGARDS. Approaches to defining incident cognitive impairment and cognitive change, including definitions employed for case/cohort studies using stored blood samples, will be described. We will discuss psychometric and methodological considerations for characterization of risks for cognitive impairment across race and region, as well as longitudinal trajectories of cognitive function.

